# Diagnostic Accuracy of Quantitative Imaging Biomarkers in the Differentiation of Benign and Malignant Vertebral Lesions: Combination of Diffusion-Weighted and Proton Density Fat Fraction Spine MRI

**DOI:** 10.1007/s00062-021-01009-1

**Published:** 2021-03-31

**Authors:** Frederic Carsten Schmeel, Simon Jonas Enkirch, Julian Alexander Luetkens, Anton Faron, Nils Lehnen, Alois Martin Sprinkart, Leonard Christopher Schmeel, Alexander Radbruch, Ulrike Attenberger, Guido Matthias Kukuk, Petra Mürtz

**Affiliations:** 1 Department of Neuroradiology, University Hospital Bonn, Rheinische-Friedrich-Wilhelms-Universität Bonn, Venusberg-Campus 1, 53127 Bonn, Germany; 2 Research Group Clinical Neuroimaging, German Centre for Neurodegenerative Diseases (DZNE), Bonn, Germany; 3 Department of Radiology, University Hospital Bonn, Rheinische-Friedrich-Wilhelms-Universität Bonn, Bonn, Germany; 4 Department of Radiotherapy and Radiation Oncology, University Hospital Bonn, Rheinische-Friedrich-Wilhelms-Universität Bonn, Bonn, Germany; 5 Department of Radiology, Cantonal Hospital Graubuenden, Chur, Switzerland

**Keywords:** Chemical-shift imaging, Diffusion magnetic resonance imaging, Bone marrow neoplasms, Spinal fractures, Fat quantification

## Abstract

**Purpose:**

To compare and combine the diagnostic performance of the apparent diffusion coefficient (ADC) derived from diffusion-weighted imaging (DWI) and proton density fat fraction (PDFF) derived from chemical-shift encoding (CSE)-based water-fat magnetic resonance imaging (MRI) for distinguishing benign and malignant vertebral bone marrow lesions (VBML).

**Methods:**

A total of 55 consecutive patients with 53 benign (traumatic, inflammatory and primary) and 36 malignant (metastatic and hematologic) previously untreated VBMLs were prospectively enrolled in this IRB-approved study and underwent sagittal DWI (single-shot spin-echo echo-planar with multi-slice short TI inversion recovery fat suppression) and CSE-based MRI (gradient-echo 6‑point modified Dixon) in addition to routine clinical spine MRI at 1.5 T or 3.0 T. Diagnostic reference standard was established according to histopathology or imaging follow-up. The ADC = ADC (0, 800) and PDFF = fat / (water + fat) were calculated voxel-wise and examined for differences between benign and malignant lesions.

**Results:**

The ADC and PDFF values of malignant lesions were significantly lower compared to benign lesions (mean ADC 861 × 10^−6^ mm^2^/s vs. 1323 × 10^−6^ mm^2^/s, *p* < 0.001; mean PDFF 3.1% vs. 28.2%, *p* < 0.001). The areas under the curve (AUC) and diagnostic accuracies were 0.847 (*p* < 0.001) and 85.4% (cut-off at 1084.4 × 10^−6^ mm^2^/s) for ADC and 0.940 (*p* < 0.001) and 89.9% for PDFF (cut-off at 7.8%), respectively. The combined use of ADC and PDFF improved the diagnostic accuracy to 96.6% (malignancy if ADC ≤ 1118.2 × 10^−6^ mm^2^/s and PDFF ≤ 20.0%, otherwise benign).

**Conclusion:**

Quantitative evaluation of both ADC and PDFF was useful in differentiating benign VBMLs from malignancy. The combination of ADC and PDFF improved the diagnostic performance and yielded high diagnostic accuracy for the differentiation of benign and malignant VBMLs.

## Introduction

The spine is the most common site of osseous metastasis in the body. In autopsy studies almost 40% of patients with metastatic solid primary tumors showed a spinal tumor manifestation, with an estimated 10% of cancer patients developing symptomatic bone metastases during the course of the disease [1]. These patients are at risk for significant comorbidities, such as pain, pathological fractures and compression of the spinal cord [2]. An accurate diagnosis of benign and malignant vertebral bone marrow lesions (VBMLs) is thus essential to enable proper clinical staging and treatment planning.

Magnetic resonance imaging (MRI) plays a central role in the diagnostic work-up and preliminary etiologic characterization of VBMLs. Routinely used anatomic MRI sequences can provide a variety of signal intensity and morphologic patterns that may allow VBMLs to be distinguished from normal bone marrow with high diagnostic accuracy [3]; however, distinguishing between different entities of benign and malignant VBMLs can be more complex as the corresponding MRI signal characteristics may overlap, leading to misidentification of malignant VBMLs in 6–21% of cases [4]. This is reflected by the highly variable diagnostic value of conventional MRI sequences for differentiating between benign and malignant VBMLs, which has been reported in various studies for sensitivity and specificity with values of 42–100% and 52–100%, respectively [5]. Establishing a definitive diagnosis of VBMLs can thus become a diagnostic challenge, especially if there is no involvement of the paravertebral soft tissues in the early stages of malignant disease.

Numerous parametric MRI techniques, which reflect particular aspects of the pathophysiology and microenvironment of vertebral lesions, have been proposed to further improve the diagnostic accuracy in distinguishing benign from malignant VBMLs, including diffusion-weighted imaging (DWI) and chemical-shift encoding (CSE)-based water-fat MRI [6]. Quantitative evaluation of the apparent diffusion coefficient (ADC) derived from DWI has been traditionally used in musculoskeletal oncology to aid the distinction of benign bone marrow edema from tumorous infiltration [7]. Malignant medullary processes usually show lower ADC values than focal benign VBMLs and osteoporotic vertebral body fractures, likely due to increased cellularity in malignant lesions [8]. More recently, CSE-based water-fat MRI has emerged as a promising imaging biomarker to assess the etiology of different types of VBMLs and compression fractures of the spine [9, 10]. Malignant VBMLs are generally associated with the replacement of bone marrow adipose tissue by cancer cells, with a subsequent decrease of the measured proton density fat fraction (PDFF) in affected vertebral bodies [11]. Numerous validation studies have shown excellent accuracy of PDFF for determining the bone marrow fat content by using either the histopathologically determined fat content in human bone samples [12] or magnetic resonance spectroscopy-based fat fraction estimations of spine marrow as the reference standard [13].

Although attempts have been made to assess the role of quantitative DWI or PDFF in differentiating benign from malignant vertebral lesions, it remains largely unclear how the diagnostic accuracy of ADC compares with the diagnostic accuracy afforded by PDFF. Moreover, to the best of our knowledge, the discriminatory ability of the combined use of ADC and PDFF measurements in order to differentiate between benign and malignant VBMLs has not yet been evaluated. Therefore, this study was set out to intraindividually compare and combine the diagnostic performance of ADC and PDFF in the differentiation of benign and malignant VBMLs.

## Material and Methods

All procedures performed in studies involving human participants were in accordance with the ethical standards of the institutional and/or national research committee and with the 1964 Helsinki Declaration and its later amendments or comparable ethical standards. Institutional review board approval for this prospective intraindividual diagnostic study (approval no. 177/15, Medical Faculty, University of Bonn) and written informed consent from all study participants were obtained prior to evaluation.

### Subjects

In this study 55 consecutive patients (25 men, mean age 68 ± 14 years) referred to spine MRI were prospectively enrolled from June 2018 to September 2019. Inclusion criteria were presence of at least one VBML with ≥ 1 cm in size as determined on routine clinical spine MRI or at least one of the following indications: a) clinically suspected acute vertebral fracture and acute onset of back pain (i.e., ≤ 1 month from admission), b) suspected osseous metastasis or malignant spine disease, and/or c) persisting localized back pain without typical discogenic radiation for more than 3 months. Three patients without bone marrow abnormalities were additionally included for quantitative analysis of healthy bone marrow. To establish a diagnostic standard of reference, further inclusion criteria were either histological confirmation of VBMLs or, if histopathology could not be obtained, follow-up MRI of at least 6 months and/or additional imaging examinations of the spine segment under investigation. Apart from general contraindications to MRI (e.g., nonconditional cardiac pacemaker), exclusion criteria included previous or concurrent chemotherapy (including angiogenesis inhibitors) and/or radiotherapy, bisphosphonate and/or growth colony-stimulating factor treatment, previous surgery and metallic implants in the spine segment under investigation.

### MR Imaging Protocol

MR imaging was performed on clinical whole-body 1.5 T and 3.0 T systems (Ingenia, Philips Healthcare, Best, The Netherlands; for 1.5 T: gradient system: 45 mT/m maximum amplitude, 200 T/m/s maximum slew rate; for 3.0 T: 80 mT/m maximum amplitude, 200 T/m/s maximum slew rate; equipped with dual-source RF transmission technology). Morphological MR imaging of the spine was acquired according to the routine clinical MRI protocol used at our institution which included at least a sagittal T1-weighted spin-echo (450–750/6–12, repetition time, TR, msec/echo time, TE, msec) and T2-weighted turbo spin-echo sequence (3000–5000/80–120, TR/TE) as well as a sagittal T2 spectral attenuated inversion recovery (SPAIR)-weighted turbo spin-echo sequence (3000–5000/50–120, TR/TE). In patients with suspected osseous metastasis or malignant disease, morphological imaging included an additional contrast-enhanced T1-weighted spin-echo sequence performed in sagittal orientation after i.v. administration of a Gd-DO3A-butrol (Gadovist, Bayer, Leverkusen, Germany) based contrast agent. Field of view, matrix size, slice thickness, and interslice gap were tailored to the specific site under study. Choice of coils was also dependent on the specific anatomic site using either the posterior coil and/or total spine coil.

A single-shot spin-echo type echo-planar DWI sequence with multislice short TI inversion recovery (STIR) fat suppression at TI = 180 ms/260 ms (1.5T/3.0 T) and four b‑values (0, 50, 200, 800 s/mm^2^) was acquired in the sagittal plane before contrast agent administration. Isotropic (directionally independent) diffusion-weighted images were reconstructed from the images with diffusion-sensitized gradients in three orthogonal directions on the MRI system. The sequence parameters at 1.5T/3.0 T were as follows: TE = 45/45 ms; TR = 3500/4300 ms; averages = 3/3 for the low b‑values and 6/6 for maximum b‑value acquisition matrix = 116 × 58 mm /116 × 58 mm; slice thickness = 4.5 mm /4.5 mm; number of slices = 18/18 (no gap); field-of-view (FH × AP × RL) = 350 × 180 × 81 mm /350 × 180 × 81 mm; flip angle = 90°/90°; half-scan factor = 0.6/0.6; EPI-factor = 59/39; parallel imaging with sensitivity encoding (SENSE) factor = 3/3; voxel size = 3 × 3.1 mm/3 × 3.1 mm acquisition time = 3:06min/2:22 min. Parametric ADC maps were calculated offline using MATLAB (MathWorks, Natick, MA, USA) according to ADC = (ln(S(0)) − ln(S(b = 800)) / 800 with S(b) and S(0) signal intensities with and without motion probing gradients.

To determine the relative PDFF fat / (water + fat), a 3D spoiled gradient-echo modified Dixon sequence (mDIXON Quant, Philips Healthcare) was acquired in sagittal orientation. This sequence acquires six evenly spaced echoes to correct for T2* effects [14], uses a low flip angle of 3° to limit T1-bias [15] and uses a multi-peak fat modelling by incorporating a precalibrated 7‑peak fat spectrum as proposed by Yu et al. [16]. The sequence parameters at 1.5 T/3.0 T were as follows: TR = 8/8 ms; TE1 = 1.15/1.15 ms; ∆TE: 1.15/1.15 ms; averages = 1/1; acquisition matrix = 175 × 100 mm; slice thickness = 4/4 mm; number of slices = 80/80 (gap −2/−2 mm); field of view = 350 × 200 × 160/350 × 200 × 160 mm; flip angle = 5°/3°; SENSE factor = 2/2; voxel size = 2 × 2/2 × 2 mm; scan time = 0:37/0:37 min. The parametric PDFF maps were automatically calculated by the imager software (Ingenia, v5.1 and above).

### Reproducibility of Quantitative Imaging Parameters

To determine whether the normal variation of ADC and PDFF measurements between our 1.5 T and 3.0 T MR scanners is below the observed differences between benign and malignant VBMLs, lumbar spine imaging data of 22 healthy volunteers (10 men; mean age 44 ± 16 years) examined with the above mentioned DWI and CSE-based MRI protocol at our 1.5 T and 3.0 T scanners on the same day were analyzed. The CSE-based water-fat MRI data of this volunteer group had already been analyzed with respect to reproducibility as part of a previous study [17] but were measured at different vertebral locations in the current study for comparative purposes. In the present study, ADC measurements were performed within the vertebral bodies Th11-L4 by using hand-drawn regions of interest (ROI), as large as possible, on midline sagittal images. The PDFF measurements were subsequently performed in the same ROIs as used for ADC measurements. Afterwards, for analysis of the reproducibility between measurements at 1.5 T and 3.0 T, the relative precision error and percentage coefficient of variation (CV = standard deviation divided by mean) were calculated.

### Diagnosis of Vertebral Lesions

The VBMLs were classified as either benign (such as acute osteoporotic vertebral fracture or degenerative endplate changes) or malignant. Diagnostic reference standard was established on the basis of biopsy and histopathologic confirmation in 32/53 (60%) benign and 31/36 (86%) malignant VBMLs. In lesions without available biopsy, the reference standard was established according to:

1. characteristic imaging appearance in all acquired MR imaging sequences (e.g., signal abnormalities in vertebral bone marrow on T1w, T2w, T2 SPAIR and contrast-enhanced T1w MR images, if applicable),
2. typical MR imaging appearance in at least one follow-up examination of at least 6 months,
3. and/or characteristic imaging appearance in additional imaging studies including computed tomography (CT) or positron emission tomography/computed tomography (PET/CT).

Imaging criterion standards were determined by consensus reading of 2 board-certified investigators with more than 8 years and 15 years experience in interpreting MR imaging studies of the spine under full consideration of all acquired images including follow-up and/or additional imaging examinations. The VBMLs were classified as either benign or malignant according to the diagnostic standard of reference.

### Study Cohort

Of the 55 patients, 40 had benign VBMLs (acute vertebral fractures, erosive degenerative endplate changes, atypical hemangioma, acute and chronic spondylodiscitis and notochordal cell tumor), 15 had malignant VBMLs (metastases, pathological vertebral fractures from non-Hodgkin lymphoma and metastases, multiple myeloma and acute myeloid leukemia). Three patients without focal or diffuse VBMLs were additionally included for analysis of healthy bone marrow. Of the ROIs 7 (8%) were located in the cervical spine, 42 (47%) in the thoracic spine and 40 (45%) in the lumbar spine; 73 and 16 lesions were examined at 1.5T and 3 T, respectively. The primary tumors in 23 metastatic lesions and 4 pathologic vertebral fractures included prostate cancer (*n* = 11), breast cancer (*n* = 7), lung cancer (*n* = 6), renal cancer (*n* = 1), B‑cell non-Hodgkin lymphoma (*n* = 1) and chronic lymphatic leukemia (*n* = 1). Histopathologic confirmation of malignant VBMLs was available in 12/15 patients with a total of 31 lesions, 3 patients with 5 malignant lesions were diagnosed on the basis of MRI follow-up and additional CT imaging studies, with 2 undergoing additional PET/CT imaging. Histopathology was obtained in 26/39 patients with 32 benign lesions, 18 of whom had acute benign (osteoporotic) vertebral fractures (*n* = 24), 3 had atypical hemangiomas, 3 had acute spondylodiscitis, 1 had a benign notochordal cell tumor and 1 had chronic abscessed spondylodiscitis. In the remaining 14 patients with 21 benign lesions without biopsy, diagnosis was based on MRI follow-up and/or additional imaging studies: 6 patients with acute vertebral fractures (*n* = 13) underwent MRI follow-up and additional CT imaging; 3 patients with acute spondylodiscitis underwent additional CT imaging, of whom 2 had additional PET/CT imaging and 1 had MRI follow-up; 2 patients with erosive degenerative endplate changes underwent additional CT imaging; 3 patients with atypical hemangioma underwent MRI follow-up, 2 of whom had additional CT imaging. There was no statistically significant difference between the benign and malignant group regarding both patient age and gender distribution (each *p* > 0.05).

### Image Analysis

Qualitative image analyses were performed by a board-certified radiologist with 8 years experience in interpreting spine MRI. VBMLs were rated as malignant or benign based on the routine clinical spine MRI protocol described above, with knowledge of the clinical patient history to reflect the situation in clinical routine. Diagnostic confidence of VBML classification was reported as uncertain in cases short-term follow-up imaging or a biopsy was suggested for further verification in the clinical setting.

Quantitative image analyses were performed in consensus by a board-certified radiologist with 7 years experience in interpreting spine MRI, and a PhD level physicist with more than 20 years experience in DWI. Both were blinded to patient-related information. Morphological imaging sequences and both ADC and PDFF maps were cross-linked to ensure correct lesion detection and delineation. For each VBML, freehand ROIs were placed at a single slice with the largest possible lesion diameter within the area of hyperintense bone marrow signal on DW images and copied onto the corresponding ADC and PDFF maps. If no abnormal signal intensity on the DW image and/or corresponding ADC parameter map was observed, the ROI was placed within the area of signal abnormalities on the corresponding T1 weighted images (mild osteoporotic vertebral fractures, *n* = 7; degenerative endplate changes, *n* = 3; acute myeloid leukemia with homogeneous appearance in DWI, *n* = 4). Areas close to the rim, with initially very low signal-to-noise ratio (SNR) or containing intralesional hemorrhage were excluded from measurement (if possible). In patients without malignant disease, freehand ROIs were placed in up to 12 different vertebral bodies per patient, as large as possible, on a central slice. For each ROI, a mean parameter value was determined for ADC and PDFF.

### Statistical Analysis

Statistical analysis was conducted using SPSS (Version 24.0, IBM, Chicago, IL, USA) and pROC package in R [18]. Mean ± standard deviation was calculated for all applicable clinical and imaging data, unless otherwise specified. Statistical significance (*p* < 0.05) was tested using Wilcoxon signed-rank test for dependent samples and Mann–Whitney U‑test for independent samples. A pairwise comparison was performed between 1.5T and 3.0 T data of the volunteer group. Group comparisons were performed for benign and malignant VBMLs, benign VBMLs and normal bone marrow, and for malignant and normal bone marrow. In order to differentiate benign and malignant VBMLs, receiver operating characteristic (ROC) analysis was performed for ADC, PDFF, and a combination of ADC and PDFF obtained by binary logistic regression (Combination(ADC, PDFF)). Optimal cut-off points for differentiation of benign and malignant VBMLs were selected for each parameter (ADC, PDFF, or Combination (ADC, PDFF)) according to maximum Youden index. The DeLong method was used to compare the dependent ROC curves [19]. Furthermore, to facilitate clinical application, a more practical approach with suitable cut-off points for simultaneous use of the two single parameters ADC and PDFF were determined. Hereby, the PDFF cut-off value was optimized for maximum sensitivity (all malignant lesions below the cut-off value, healthy bone marrow above). The ADC threshold was then determined to optimally differentiate between the benign and malignant lesions with a PDFF above the cut-off value (maximum Youden index).

A priori statistical power analyses revealed that 36 observations per group would provide sufficient power (β = 0.9) to show a significant difference at an alpha error of 0.05, assuming that the majority of all malignant VBMLs would show lowered ADC and PDFF values as a result of tumorous infiltration (estimated probability 1:0.8) [20].

## Results

### Qualitative Reader Performance

The clinical reader correctly diagnosed 41/53 VBMLs as benign and 35/36 VBMLs as malignant, whereas 12 benign lesions were incorrectly rated as malignant (1 benign notochordal cell tumor, 5 osteoporotic fractures, 3 atypical hemangioma, 3 spondylodiscitis) and 1 malignant lesion (pathologic fracture) was incorrectly diagnosed as being benign. The corresponding diagnostic performance was as follows: AUC 0.873; sensitivity 97.22%; specificity 77.36%; positive predictive value (PPV) 74.47%; negative predictive value (NPV) 97.62%; accuracy 85.39%. Uncertain findings clinically prompting short-term follow-up or biopsy were reported for 4 acute osteoporotic fractures (rated falsely positive) and 2 spondylodiscitis (rated falsely positive).

### Reproducibility of Quantitative Imaging Parameters at 1.5T and 3.0 T

In the 22 healthy volunteers, a total of 150 ROIs were analyzed within the vertebral bodies Th11-L4. The ADC measurements at our 1.5 T and 3.0 T scanners amounted to 736 ± 99 × 10^−6^ mm^2^/s and 708 ± 163 × 10^−6^ mm^2^/s, respectively and PDFF values at 1.5 T and 3.0 T were 46.4 ± 11.1% and 45.8 ± 12.4%, respectively. According to Wilcoxon test, the differences of the ADC values at 1.5 T and 3.0 T were on average 27.6 × 10^−6^ mm^2^/s and statistically not significant (*p* = 0.061), whereas differences for PDFF were statistically significant (*p* = 0.001), but on average only 0.6%. The root-mean-square deviations were 154.7 × 10^−6^ mm^2^/s for ADC and 2.7% for PDFF. The mean CV of ADC and PDFF measurement amounted to 12.1 ± 10.3% and 3.2 ± 3.0%, respectively.

### Quantitative Analysis

**Fig. 1:**
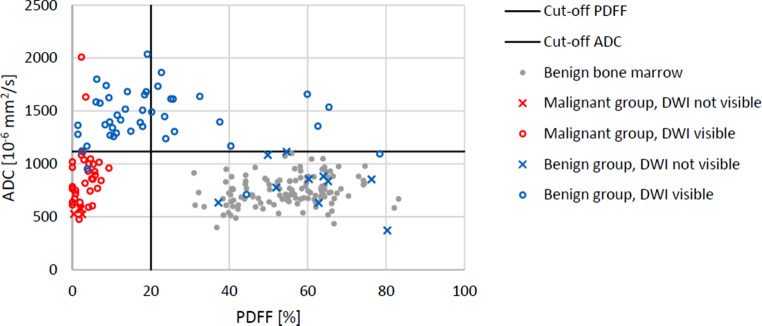
Dot chart demonstrating mean ADC (×10^−6^ mm^2^/s) and percentage PDFF measurement values obtained by ROI analysis of benign (*blue dots and crosses*) and malignant (*red dots and crosses*) VBMLs as well as benign bone marrow (*grey dots*) in our cohort. Dots represent lesions with hyperintense signal on DWI whereas crosses show lesions without signal abnormalities on DWI. Calculated quantitative cut-off values for simultaneous use of ADC (1118 × 10^−6^ mm^2^/s) and PDFF (20.0%) are indicated by the horizontal and vertical solid line, respectively

The ADC and PDFF ROI analyses were performed in a total of 53 benign and 36 malignant VBMLs as well as in 124 normal vertebral bodies (Fig. 1). Mean ADC and PDFF values for the various VBML types are summarized in Table 1. The ROI size in benign and malignant VBMLs amounted to 206 ± 105mm^2^ (range 12–390mm^2^) and 219 ± 130mm^2^ (range 18–510mm^2^), respectively.

**Table 1:** Benign and malignant VBMLs with corresponding ADC and PDFF values

Lesion type	*N*	Mean ADC ± SD	Mean PDFF ± SD
		(×10^−6^ mm^2^/s)	(%)
**Benign VBMRLs**			
Osteoporotic vertebral fracture	37	1352 ± 351	27.26 ± 20
Atypical hemangioma	6	1445 ± 241	49.88 ± 26.84
Acute spondylodiscitis	6	1342 ± 106	7.78 ± 4.35
Erosive endplate degenerative changes	2	542 ± 240	62.35 ± 25.39
Chronic spondylodiscitis	1	953	3.8
Benign notochordal cell tumor	1	1285	1.4
*Total*	*53*	*1323* *±* *349*	*28.2* *±* *23.1*
**Malignant VBMRLs**			
Metastasis	23	842 ± 297	3.27 ± 2.82
Multiple myeloma	3	1056 ± 41	4.1 ± 1.91
Metastatic pathologic vertebral fracture	4	1128 ± 367	2.78 ± 1.62
Ewing sarcoma	2	876 ± 90	3.6 ± 0.57
Acute myeloid leukemia	4	552 ± 27	1.7 ± 0.8
*Total*	*36*	*861* *±* *300*	*3.1* *±* *2.4*
**Normal bone marrow**			
*Total*	*124*	*744* *±* *136*	*54.9* *±* *11.0*

*ADC* apparent diffusion coefficient; *N* number, *PDFF* proton density fat fraction, *SD* standard deviation, *VBML* vertebral bone marrow lesion

**Fig. 2:**
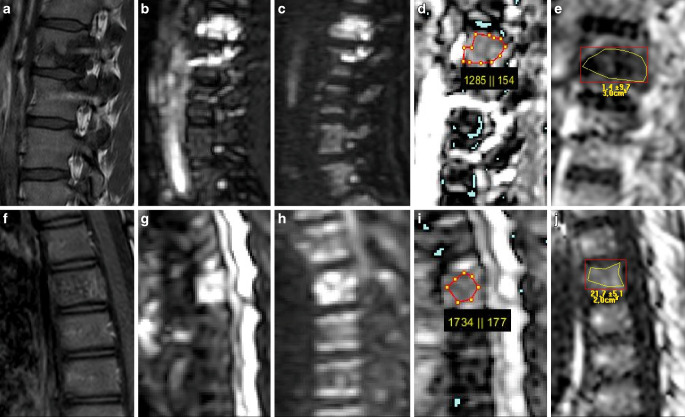
Two examples of benign VBMLs. *Upper row* (**a–e**): histopathologically confirmed benign notochordal cell tumor of the thoracic spine. *Lower row* (**f–j**): biopsy proven atypical hemangioma of the thoracic spine. Sagittal T1-weighted SE images (**a,** **f**), DW images with b = 0 s/mm^2^ (**b,** **g**) and b = 800 s/mm^2^ (**c,** **h**) and the corresponding ADC (×10^−6^ mm^2^/s) (**d,** **i**) and PDFF (%) (**e,** **j**) parameter maps with exemplary ROI measurements. ADC correctly identified the notochordal cell tumor as benign, whereas PDFF falsely classifies the lesion as malignant due to high amount of edema. Both ADC and PDFF correctly identified atypical hemangioma as benign lesion

**Fig. 3:**
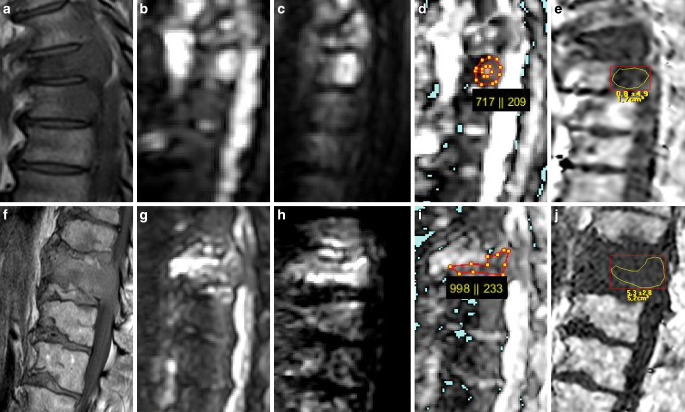
Two examples of malignant VBMLs. *Upper row* (**a–e**): histopathologically confirmed breast cancer metastasis and pathologic vertebral fracture of the thoracic spine. *Lower row* (**f–j**): biopsy proven pathologic fracture of the thoracic spine due to renal cancer metastasis. Sagittal T1-weighted SE images (**a,** **f**), DW images with b = 0 s/mm^2^ (**b,** **g**) and b = 800 s/mm^2^ (**c,** **h**) and the corresponding ADC (×10^−6^ mm^2^/s) (**d,** **i**) and PDFF (%) (**e,** **j**) parameter maps with exemplary ROI measurements. Both ADC and PDFF correctly identified metastatic lesions as malignant

**Fig. 4:**
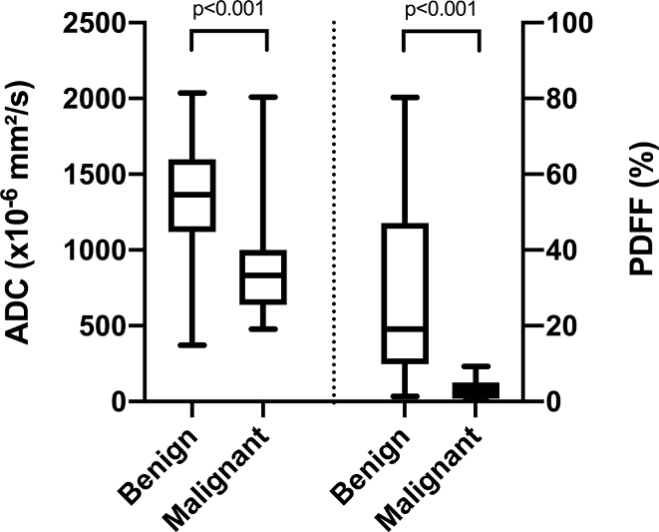
Box-and-whisker plots of benign and malignant VBMLs demonstrating summary values of mean ADC (×10^−6^ mm^2^/s) and percentage PDFF. Horizontal solid lines show minimum (*left*) and maximum (*right*) observations, respectively. Boxes represent the data between the 25th percentile and the 75th percentile. Median is shown as vertical line across each box. Malignant lesions tend to show lower ADC and PDFF values than benign bone marrow replacing processes

The ADC and PDFF values of malignant VBMLs were statistically significantly lower than those of benign VBMLs (*p* < 0.001). Example images are given in Figs. 2 and 3. The ADC and PDFF values for the 36 malignant lesions were 861 ± 300 × 10^−6^ mm^2^/s and 3.1 ± 2.4%, respectively, while ADC and PDFF for the 53 benign VBMLs were 1323 ± 349 × 10^−6^ mm^2^/s and 28.2 ± 23.1%, respectively. Subgroup ADC and PDFF values of VBMLs are graphically illustrated in Fig. 4. For normal vertebral bone marrow, the ADC and PDFF values amounted to 744 ± 136 × 10^−6^ mm^2^/s and 54.9 ± 11.0%, respectively. The PDFF values for normal vertebral bone marrow were significantly higher than for benign and malignant VBMLs (both *p* < 0.001). The ADC values for normal vertebral bone marrow were significantly lower than for benign VBMLs (*p* < 0.0001) and slightly lower than for malignant VBMLs (*p* = 0.032).

### Diagnostic Performance of Quantitative Parameters

**Fig. 5:**
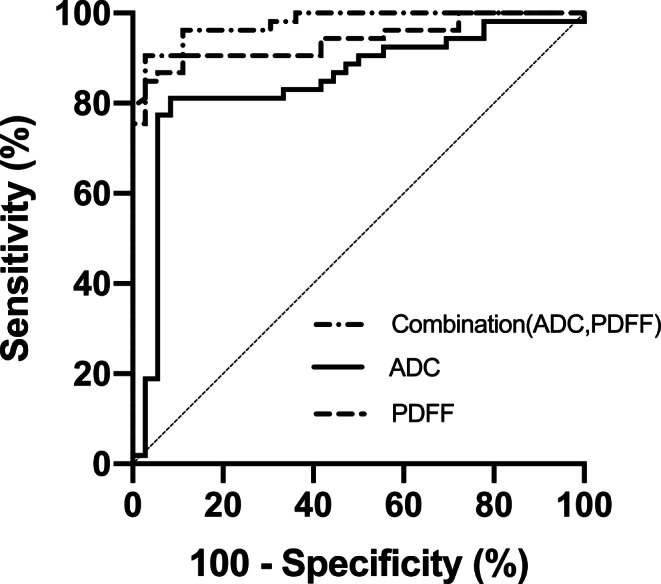
Receiver operating characteristic (ROC) curves of the quantitative imaging parameters ADC, PDFF and Combination(ADC, PDFF) for the differentiation between benign and malignant VBMLs

Results of ROC analysis are given in Table 2 and Fig. 5. By the combined parameter Combination(ADC, PDFF) obtained by binary logistic regression, the probability of malignancy was $1/(1+\exp (-\mathrm{S}))\text{with}\mathrm{S}=-7.711+0.326\cdot \text{PDFF}+0.005\cdot \mathrm{ADC}$. The AUC for ADC, PDFF, and Combination(ADC, PDFF) were 0.847, 0.940, and 0.977, respectively. For the single parameters ADC and PDFF, and the Combination(ADC, PDFF), cut-off values of 1084.4 × 10^−6^ mm^2^/s, 7.8%, and 0.204, respectively, were best suited to differentiate between benign and malignant vertebral lesions, whereby 85.4%, 89.9%, and 93.3% of the VBMLs were correctly identified as malignant or benign. Comparing the individual AUC of the single parameters ADC and PDFF, and of the Combination(ADC, PDFF), statistically significant differences were found only between ADC and Combination(ADC, PDFF) (*p* = 0.003), whereas no statistically significant differences were observed between ADC and PDFF (*p* = 0.081) and between PDFF and Combination(ADC, PDFF) (*p* = 0.072).

**Table 2:** Diagnostic performance of ADC, PDFF and Combination(ADC, PDFF) for differentiating benign from malignant VBMLs

Parameter	AUC	SE	*P*	CI1	CI2	Cut-off	Sen	Spec	Acc
*ADC*^*a*^	0.847	0.045	<0.001	0.758	0.936	1084.4	0.917	0.811	0.854
*PDFF*^*a*^	0.940	0.025	<0.001	0.891	0.989	7.8	0.972	0.849	0.899
*Combination(ADC, PDFF)*	0.977	0.012	<0.001	0.953	1.000	0.204	0.972	0.906	0.933

Combination(ADC, PDFF) was obtained by binary logistic regression analysis. The probability of malignancy was $1/(1+\exp (-\mathrm{S}))\text{with}\mathrm{S}=-7.711+0.326\cdot \text{PDFF}+0.005\cdot \mathrm{ADC}$. The optimal cut-off point of each ROC analysis was selected according to maximum Youden index. Cut-off points are given in units of 10^−6^ mm^2^/s for ADC and of % for PDFF

*AUC* area under the curve, *SE* standard error, *P* significance level, *CI* 95% confidence interval, *Sen* sensitivity (true positive rate), *Spec* specificity (true negative rate), *Acc* accuracy (rate of correctly identified cases)

^a^Means that a lower test result indicates a more positive test

For ADC, 10 lesions were false positive (8 osteoporotic vertebral fractures and 2 erosive degenerative endplate changes) and 3 lesions were false negative (1 pathologic vertebral fracture, 1 necrotic lung cancer metastasis, and 1 multiple myeloma). This resulted in a PPV of 76.7%, a NPV of 93.5% and an accuracy of 85.4% in distinguishing benign from malignant VBMLs. For PDFF, 8 lesions were false positive (1 benign notochordal cell tumor, 4 osteoporotic vertebral fractures, 2 acute spondylodiscitis and 1 chronic abscessed spondylodiscitis) and 1 lesion was false negative (pathologic fracture due to prostate cancer). This yielded a PPV of 81.4%, a NPV of 97.8% and an accuracy of 89.9%. With Combination(ADC, PDFF), 35 of 36 lesions were correctly classified as malignant and 48 of 53 lesions were correctly diagnosed as benign, whereas five benign lesions (1 benign notochordal cell tumor, 1 acute osteoporotic vertebral fracture, 2 acute spondylodiscitis and 1 chronic abscessed spondylodiscitis) were incorrectly classified as malignant and one malignant lesion (necrotic lung cancer metastasis) was incorrectly classified as benign. This yielded a PPV of 87.5%, an NPV of 98.0% and an accuracy of 90.6% for the differentiation of benign and malignant VBMLs.

In an effort to evaluate a more practical approach for routine clinical application, the simultaneous use of the two single parameters PDFF and ADC was tested. The cut-off point for PDFF was determined by ROC analysis of malignant VBMLs and normal bone marrow resulting in 20.0%. The cut-off point for ADC was determined by ROC analysis of malignant and benign VBMLs resulting in 1118.2 × 10^−6^ mm^2^/s. With the combined use of these two cut-off points (malignancy, if values are both smaller than cut-off points, otherwise benignity) lesions were classified with a sensitivity, specificity and accuracy of 94.6%, 98.1% and 96.6%, respectively. One benign lesion (chronic abscessed spondylodiscitis) was incorrectly classified as malignant whereas two malignant VBMLs (1 pathological fracture due to chronic lymphatic leukemia, 1 necrotic lung cancer metastasis) were falsely classified as benign. This yielded a PPV of 97.2% and a NPV of 96.2% for differentiation of benign and malignant VBMLs.

In the VBML subgroup reported to be uncertain by the clinical reader, utilizing the 1084.4 × 10^−6^ mm^2^/s ADC cut-off resulted in correct diagnosis of 5/6 acute osteoporotic fractures and 2/2 spondylodiscitis. Utilizing the 7.8% PDFF cut-off resulted in correct diagnosis of 6/6 osteoporotic fractures and 2/2 spondylodiscitis. Utilizing simultaneous use of 1118.2 × 10^−6^ mm^2^/s ADC cut-off and 20% PDFF cut-off also resulted in correct diagnosis of the clinically uncertain VBMLs.

## Discussion

This prospective study evaluated the diagnostic performance of quantitative ADC and PDFF measurements to differentiate benign from malignant VBMLs. The major finding revealed that there are statistically significant differences in ADC and PDFF values between benign and malignant VBMLs, allowing for a differentiation of benign and malignant entities with high diagnostic accuracy. By combined use of ADC and PDFF at cut-off points of 1118 × 10^−6^ mm^2^/s and 20.0%, respectively, the diagnostic accuracy could be significantly improved compared to the either single quantitative parameters (96.6% versus 89.9% for PDFF and 85.4% for ADC). Owing to the excellent accuracy of the combined use of ADC and PDFF, our data strongly suggest that additional imaging studies and potentially harmful bone biopsies can often be avoided in patients with indeterminate VBMLs, with the potential advantage of improving overall patient health care and associated costs.

One retrospective study has assessed the diagnostic accuracy of single ADC and PDFF for differentiation of metastases and benign bone marrow abnormalities using a critical cut-off value of ≤ 995 × 10^−6^ mm^2^/s for ADC and ≤ 9% for PDFF [21]. Although the diagnostic performance of ADC and PDFF was not statistically compared in that study, the authors concluded that PDFF is superior to ADC in order to differentiate focal benign VBMLs and metastases. Compared to the present work, the accuracy in that study was higher for PDFF (94.3% compared to 89.9%), but lower for ADC (80.7% compared to 85.4%). More recently, Donners et al. have compared the diagnostic accuracy of ADC and fat fraction derived from a 2-point Dixon sequence for differentiation of osteoporotic and malignant vertebral fractures at calculated cut-off values of ≤ 1040 × 10^−6^ mm^2^/s for ADC and ≤ 11.5% for PDFF [22]. Besides the fact that fat fraction showed higher diagnostic accuracy than ADC (87% vs. 76%) for distinguishing benign from malignant vertebral fractures, that study also demonstrated that both ADC and fat fraction were capable of improving the diagnostic accuracy, especially specificity, of two independently evaluating radiologists with different levels of expertise, which is in agreement with our observations in VBMLs reported to be uncertain on routine spine MRI. We also found a slight, although not statistically significant diagnostic superiority of PDFF over ADC with respect to the corresponding ROC curves. Additionally, our analysis showed that DWI has some inherent diagnostic limitations in cases of either low-grade or non-edematous benign lesions (such as mild compression fractures or diffuse hematologic neoplasms) or predominantly necrotic malignant lesions, whereas PDFF may incorrectly classify heavily sintered osteoporotic fractures and predominantly fluid-containing lesions as malignant. The overall distinction between normal bone marrow and malignant lesions was better with PDFF, whereas the etiologic characterization of benign VBMLs was better using ADC. The combined simultaneous use of the two single parameters ADC and PDFF at cut-off values of 1118.2 × 10^−6^ mm^2^/s for ADC and 20% for PDFF had the capacity to largely overcome these limitations of either single quantitative imaging parameters, resulting in only one false positive and two false negative findings. Although these results are promising, they cannot yet qualify the approach for stand-alone use, but are primarily intended to support diagnostics in combination with other dedicated anatomical sequences in the clinical setting.

Diffusivity is reduced in tissue with high cellularity, e.g. in bone marrow replaced by tumor cells, due to a reduction of the fluid component within the interstitial space. Consequently, malignant VBMLs usually present with low ADC values, whereas benign lesions show less reduction in diffusivity [7]. In a large-scale meta-analysis of previously published data, benign VBMLs demonstrated significantly higher ADC values (range 1200–2000 × 10^−6^ mm^2^/s; mean, 1679 ± 531 × 10^−6^ mm^2^/s) than malignant lesions (range 700–1300 × 10^−6^ mm^2^/s; mean, 913 ± 354 × 10^−6^ mm^2^/s), irrespective of b‑value combination and field strength [8]. With a mean ADC of 1323 ± 349 × 10^−6^ mm^2^/s for benign and 861 ± 300 × 10^−6^ mm^2^/s for malignant VBMLs in our study, the observed ADC values were consistent with those previous findings. Compared with normal bone marrow, the ADC of benign lesions was also significantly larger, while the ADC of malignant lesions was only slightly increased. The discriminatory ability of DWI to differentiate between benign and malignant entities of VBMLs yielded an accuracy of 85.4%, which corroborates the results of another recent meta-analysis [23]; however, there was certain overlap in the range of ADC values resulting in 10 false positive and 3 false negative findings. This yielded a relatively low specificity of 81.1%. According to Maeda et al. [24], three possible explanations may account for the false negative observations: necrotic tumor tissue, a large amount of associated interstitial edema and an increased perfusion fraction in the hypervascular portion of the tumor. To overcome these limitations to the best possible extent in our study, freehand ROIs were placed at the site of maximum hyperintensity observed on the b = 800 s^2^/mm DW images. In addition, the avoidance of artifacts, necrosis (if possible) and hemorrhage as determined in conjunction with morphologic MR imaging should have enhanced the reliability of the imaging data. The presence of only slight edema in benign lesions and the strong diffusion restriction in one predominantly abscessed chronic spondylodiscitis led to the false positive findings in our cohort.

Histopathologically, malignant processes are associated with the replacement of normal bone marrow fat by cancer cells whereas benign lesions usually contain at least residual amounts of adipose tissue pervaded within the bone marrow matrix [25]. Older studies, most of which used semi-quantitative in-phase/opposed-phase chemical-shift imaging, have already shown that low microscopic fat content in VBMLs may indicate malignancy [26, 27]. With recent technical advances, various CSE-based water-fat MRI techniques, e.g. the modified Dixon (mDIXON) method, have been established to quantitatively assess tissue fat content [11, 28]. These techniques have the advantage over conventional chemical-shift imaging that they correct for several confounding factors (including T1 bias, T2* effects and the multispectral complexity of the fat signal) and thus provide highly accurate measurements of tissue fat fraction [6]. The few available studies using CSE-based water-fat MRI for differentiating benign from malignant VBMLs have shown a very high accuracy of PDFF with AUCs ranging from 0.93 to 0.98 [9, 29, 30]. With an AUC of 0.94 and a cut-off value of 7.8% PDFF in our study, the diagnostic performance was on a similarly high level; however, our calculated cut-off value for the single use of PDFF was slightly higher than previously reported, which is probably due to the different composition of the study groups and inclusion of vertebral fractures and hematologic malignancies. It has been shown that hematologic neoplasms do not necessarily translate into decreased bone marrow fat fraction [31]. Another possible reason for the observed variability of the calculated PDFF cut-off value compared to previous studies is the natural physiological variation in bone marrow fat content of the spine, as there usually is a gradient of increasing fat values from the cervical to the lumbar spine and with increasing age [32, 33]. In our cohort, one pathologic fracture caused by prostate cancer metastasis with a corresponding PDFF of 9% was incorrectly classified as benign, whereas 8 benign VBMLs were incorrectly classified as malignant, which was probably due to extensive edema and/or cell debris in the affected vertebral bodies.

Apart from evaluating the diagnostic performance, an important issue in quantitative MRI is the reproducibility of the obtained imaging data which may depend on various critical factors including system stability and tumor pathophysiology itself. To the best of our knowledge, no previous study has evaluated the reproducibility of ADC measurements in vertebral bone marrow across different field strengths. This study provides evidence for a good in vivo reproducibility of ADC measurements in lumbar bone marrow across 1.5 T and 3.0 T. Particularly, the difference between the ADC reproducibility measurements was much smaller than the observed differences between benign and malignant VBMLs. Therefore, the level of agreement for ADC measurements appears clinically acceptable to detect relevant differences between benign and malignant VBMLs. The high ADC reproducibility in this study could probably not be achieved if other fat suppression methods than STIR were used. Due to the large variation of fat amount in vertebral bone marrow, it is important to ensure fat suppression, because ADC values are directly influenced by the amount of unsuppressed fat signal [34]. A slight disadvantage of using STIR fat suppression is its inherent smaller signal-to-noise ratio compared to spectral fat suppression techniques, which was sometimes critical in normal bone marrow but not in lesions. For the application of CSE-based water-fat MRI in vertebral bone marrow, numerous in vitro and in vivo studies using either water-fat phantoms [35, 36] or healthy volunteer cohorts [17, 37] have demonstrated that PDFF yields excellent precision across field strengths and reconstruction methods. Hence, there is solid evidence to suggest that PDFF is a standardized, quantitative imaging parameter [38, 39]. In line with these findings, our data demonstrated a very small relative precision error across measurements at 1.5 T and 3.0 T.

We acknowledge several limitations of this study. The sample size of our cohort was relatively small, but a priori sample size considerations indicate that sufficient statistical power is provided for the detection of clinically relevant differences. VBMLs originated from both primary bone cancer and metastases including a variety of solid primary tumors and hematologic neoplasms. This may have caused divergent measurements depending on the underlying tumor entity. Nonetheless, the differences of ADC and PDFF remained highly significant between benign and malignant entities. Image acquisition at different field strengths may have influenced the precision of the obtained quantitative imaging data; however, reproducibility analyses demonstrated only marginal average differences in ADC and PDFF measurements among our 1.5 T and 3.0 T scanners, which in most cases are likely to be clinically irrelevant for the differentiation of benign and malignant VBMLs. Further reproducibility studies are necessary to confirm these findings, and possibly substantiate the accuracy of ADC and PDFF measurements in clinical routine. Lastly, we assessed ADC and PDFF rather as a stand-alone technique to evaluate different types of VBMLs. A dedicated statistical comparison with the diagnostic accuracy of conventional MRI sequences is beyond the scope of the current study and needs to be addressed in the near future; however, two previous studies have already demonstrated that the additive use of quantitative ADC and fat fraction analyses can improve the diagnostic accuracy of radiologists using clinical routine MRI sequences to differentiate benign and malignant vertebral fractures [22] as well as vertebral hematopoietic marrow islands and metastases [40].

In conclusion, quantitative evaluation of ADC and PDFF measurements provides high diagnostic accuracy for the non-invasive differentiation of benign and malignant VBMLs. The simultaneous use of ADC and PDFF can significantly improve the differential diagnostic performance of the either single quantitative imaging parameter and might thus provide a more accurate characterization of the underlying etiology of indeterminate VBMLs; however, primarily chronic inflammatory processes may be diagnosed as falsely malignant with both ADC and PDFF, and further research with larger cohorts is needed to evaluate optimal thresholds for distinguishing inflammatory from malignant VBMLs.
